# Erratum to “Asymmetrical Transport Distribution Function: Skewness as a Key to Enhance Thermoelectric Performance”

**DOI:** 10.34133/research.1317

**Published:** 2026-06-23

**Authors:** Jin-Cheng Zheng

**Affiliations:** ^1^ Department of Physics, Xiamen University, Xiamen 361005, China.; ^2^ Department of Physics and Department of New Energy Science and Engineering, Xiamen University Malaysia, Sepang 43900, Malaysia.

In the original publication [1], Eq. (20) on page 8 was written incorrectly. The subscript of the distribution density function in the numerator should be $D_{1}$ instead of $D_{0}$. The correct equation is provided below.

$$\gamma^{\mathrm{TE}}\left(x_{0},\;b\right)=\frac{\int g_{A}\left(x,\;x_{0},\;b\right)\;D_{1}\left(x,\;x_{0},\;b\right)\;\mathrm{dx}}{\int g_{S}\left(x,\;x_{0},\;b\right)\;D_{0}\left(x,\;x_{0},\;b\right)\;\mathrm{dx}}.$$ (20)

This correction does not affect any of the subsequent analysis, discussions, or conclusions of the paper. The rest of the article remains valid as originally published.

The author apologizes for any inconvenience caused.
