# Supplementary material for: Asymmetrical Transport Distribution Function: Skewness as a Key to Enhance Thermoelectric Performance
Source: Research (Wash D C). 2022 Jul 14;2022:9867639. doi: 10.34133/2022/9867639 (PMC11734745; doi:10.34133/2022/9867639)
Supplement: Supplementary Materials — S1 thermoelectric components for the constant TDF. S2: TDF with constant location-scale family: the case of Dirac delta function. S3: TDF with typical general location-scale family: TDF in Gaussian function form. S4: parameters of Lorentzians for spectral resistivities. [file 9867639.f1.doc]

Supplemental Materials for:

**Asymmetrical transport distribution function: skewness as a key to enhance thermoelectric performance**

Jin-Cheng Zheng^(a,b)^*

(a) Department of Physics, Xiamen University, Xiamen 361005, China.

(b) Department of Physics and Department of New Energy Science and Engineering, Xiamen University Malaysia, Sepang 43900, Malaysia.

* Electronic mail: [jczheng@xmu.edu.cn](mailto:jczheng@xmu.edu.cn)

**S1. Thermoelectric components for the constant TDF.**

For the case of thermoelectrics with constant TDF, namely, *g*(*x*) = *g*_0_, the dimensionless integrals *J_n_* becomes

$J_{n}=g_{0}\int_{-\infty}^{+\infty} \text{ }D_{n}(x)dx$. (s1)

Then we have, *J*_0_ = *g*_0_, *J*_1_ = 0, and *J*_2_ = $J_{2}=\frac{\pi^{2}}{3}g_{0}$. The reduced electrical conductivity is $\sigma_{r}=g_{0}$. The reduced electronic thermal conductivity is *κ_e,r_* = $\frac{\pi^{2}}{3}g_{0}$. The Seebeck coefficient *S_r_*, power factor and the *ZT* are all zero.

**S2. TDF with constant location-scale family: the case of Dirac delta function.**

The TDF in single Dirac delta function form can be expressed as,

$g(x)=g_{0}\delta(x-x_{0})$, (s2)

where *g*_0_ is constant. This TDF is actually a Delta shape peak located at the *x* = $x_{0}$ position away from Fermi energy level. Taking advantage of *sifting property* of the delta function (the integral of the time-delayed Dirac delta),

$\int_{-\infty}^{+\infty} \text{ }f(x)\delta(x-x_{0})dx=f(x_{0})$, (s3)

one can obtain integrals *J_n_* and transport components as,

$J_{n}=g_{0}D_{n}(x_{0})$. (s4)

Thus, the reduced electric conductivity is,

$\sigma_{r}=g_{0}D_{0}(x_{0})$, (s5)

the reduced thermoelectric power (Seebeck Coefficient) becomes

$S_{r}=x_{0}$, (s6)

and the reduced power factor can be obtained as

$P_{r}=g_{0}x_{0}^{2}D_{0}(x_{0})$. (s7)

Because the reduced electronic thermal conductivity is 0,

$\kappa_{e,r}=0$, (s8)

the figure of merit (*ZT*) is finally obtained as

$ZT=g_{0}x_{0}^{2}D_{0}(x_{0})/\gamma$. (s9)

The optimal value of thermoelectric components requires different optimal location parameter *x*_0_. When the location parameter *x*_0_ = 0, the reduced electric conductivity has the highest value of σ*_r_=g*_0_/4, because the *D*_0_(*x*_0_=0) is the maximum. However, for the reduced power factor and *ZT*, the optimal value of location parameter *x*_0_ is shifted from 0. The function of $x_{0}^{2}D_{0}(x_{0})$ reaches maximum 0.44 at $x_{0}\approx$2.40, and the maximum of reduced power factor of 0.44$g_{0}$ and *ZT* of 0.44$g_{0}/\lambda$ can be obtained accordingly. After the optimization of location parameter $x_{0}$, the *ZT* can be further enhanced by the increase of scale parameter *g*_0_, or by the reduction of lattice thermal conductivity. By assuming a typical lattice thermal conductivity, *κ_l_* = 1 Wm^-1^K^-1^ at room temperature, we have *λ* = 0.0976, then the optimal *ZT* can be as high as 4.50 *g*_0_ for general Delta function as TDF. For the simpler case of TDF being the normalized Delta function (*g*_0_ =1), the *ZT* is 4.50 at room temperature, which is more than 4 times than the *ZT* of the best thermoelectric material, Bi_2_Te_3_ at room temperature. For the cases when the scale parameter *g*_0_ >1, and lattice thermal conductivity is lower than 1 Wm^-1^K^-1^, the maximum *ZT* will be even much higher (e.g., if *g*_0_ >2.23, *ZT* is higher than 10).

**S3. TDF with typical general location-scale family: TDF in Gaussian function form**

The Gaussian TDF thus can be expressed as,

$g(x)=\frac{1}{b\sqrt{2\pi}}\exp\left[-\frac{(x-x_{0})^{2}}{2b^{2}}\right]$, (s10)

The location parameter *x*_0_ defines the peak position of the Gaussian function, and the scale parameter *b* characterizes the width of the peak. The TDFs in Gaussian function form with typical location parameter (*x*_0_ = 0 and 2.4) and scale parameter (b = 0.5, 1, and 3) are shown in Fig. S1(a). The thermoelectric properties, including the reduced electrical conductivity, the reduced thermoelectric power (Seebeck coefficient), the power factor, the reduced electron thermal conductivity and the figure of merit (*ZT*) of thermoelectric material as a function of location parameters and scale parameters are shown in Fig. S1(b-f), respectively. Typical location parameters (*x*_0_ = 0, 1, 2.4 and 5) are shown to illustrate the trend of thermoelectric properties.

The Gaussian TDF represents a wide range of general distributions. Firstly, regarding the scale parameter (*b*), it represents the width of the peak or the spread of the distribution. The smaller the value of *b*, the narrower or shaper the peak; the larger the value of *b*, the flatter the peak. This feature is similar with density of states (DOS) of thermoelectric materials. For more localized orbital, the peak of DOS is narrower, and for more delocalized electrons, the associated DOS is more flat. In extreme cases, such as if the scale parameter (*b*) approaches zero, the TDF will be approaching Dirac delta function, δ(*x*). On the other hand, if the scale parameter (*b*) approaches infinite, then the TDF will be approaching constant distribution. Both extreme cases have been discussed in previous parts. Secondly, the location parameter can be associated with the position of Fermi energy at zero temperature or chemical potential at finite temperature. It is well known that the chemical potential can be tuned by doping [S1] or pressure [S2,S3] or strains [S4,S5], thus the carrier velocity, relaxation time and DOS can be modified accordingly. Subsequently, the thermoelectric transport properties are changed too. Therefore, if the scale parameter (*b*) reflects the peak width of TDF (mainly related to electronic structure features such as DOS and its spread associated with temperature through Fermi-Dirac distribution), the location parameter characterizes the shifting the chemical potential due to chemical pressure (such as impurity or defects) or mechanical pressure (including hydrostatic pressure or strain fields).


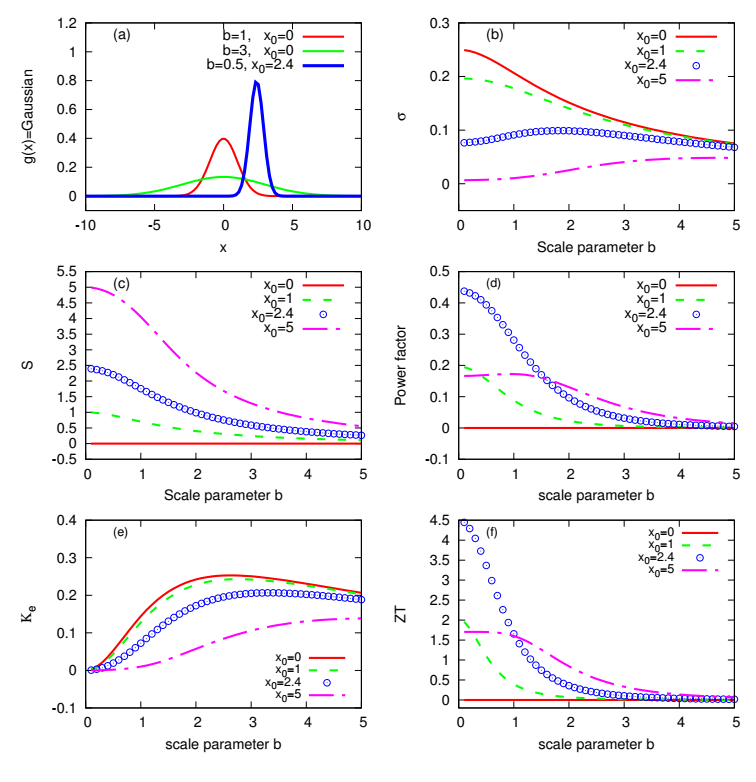


Fig. S1. The thermoelectric properties of system with TDF in a Gaussian function form. (a) The transport density function (TDF) in a Gaussian function form. Examples of the location parameter (*x*_0_=0 and 2.4) and scale parameter (*b=* 0.5, 1, and 3) are shown in the figure. The reduced electrical conductivity (b), the reduced thermoelectric power (Seebeck coefficient) (c), the power factor (d), the reduced electron thermal conductivity (e) and the figure of merit (*ZT*) (f) of thermoelectric material as a function of location parameters and scale parameters. Typical location parameters (*x*_0_=0, 1, 2.4 and 5) are presented.

From Fig. S1, it can be found that the response of thermoelectric components to location parameter and scale parameter are significantly different. The electrical conductivity and the electron thermal conductivity decrease with the increase of location parameter *x*_0_, but the thermoelectric power increases with increase of location parameter. When the location parameter *x*_0_ = 0, the mean, median and mode of the Gaussian distribution are located at 0. The electrical conductivity and the electron thermal conductivity reaches its largest value, while for the thermoelectric power (Seebeck coefficient, S), it is just the opposite, and it becomes zero, independent on scale parameter. Therefore, other thermoelectric quantities, which are proportional to thermoelectric power (S), such as the power factor, and *ZT*, are all zero for the case of the location parameter *x*_0_ = 0, as shown in solid line in red color in Fig. S1 (c, d, and f). This is very similar with (or approximately close to) the case of good metal in real materials. For a good metal, the DOSs located at the Fermi energy (or chemical potential at finite temperature) are normally quite large, which can be very close to Gaussian TDF with location parameter near zero. The trends of the electrical conductivity and the electron thermal conductivity as well as the thermoelectric power in terms of location parameters well explain that the normal good metals have very good electrical conductivity and electron thermal conductivity, but have very poor thermoelectric power.

For semiconducting materials, the DOS may have several peaks, and the peak positions are normally away from the Fermi energy depending on doping level. This case is similar with the Gaussian TDF with non-zero location parameter and finite scale parameter. From Fig. S1, one can see that with the increase of location parameter, the electrical conductivity reduces but the thermoelectric power increase. It makes the trend of power factor and *ZT* varies more complicated with location parameter and scale parameter, because the power factor and *ZT* are strongly coupled with both electrical conductivity and thermoelectric power. The overall absolute value of power factor or *ZT* at larger scale parameter are very small although they will be slightly enhanced by increasing location parameter. This is because Gaussian TDF with large scale parameter will be closer to constant TDF, and as we discussed in previous part, the electrical conductivity can be maintained in certain level, but the thermoelectric power, power factor and thus the *ZT* are becoming small. To enhance the *ZT*, the scale parameter should be reduced and the value of location parameter should be optimized. As shown in Fig. S1, the optimal location parameter is about *x*_0_ = 2.4, the power factor and *ZT* are significantly enhanced with smaller scale parameter, and they reach the maximum when the scale parameter approaches zero. That is exactly the case of TDF being Dirac delta function.

**S4. Parameters of Lorentzians for spectral resistivities.**

Table S1. Parameters of Lorentzians for spectral resistivities, values given by Landauro et al [S6].

| Materials | A | $x_{01}$ | $b_{1}$ | *α* | $x_{02}$ | $b_{2}$ |
| --- | --- | --- | --- | --- | --- | --- |
|  | (μΩ cm eV) | (eV) | (eV) |  | (eV) | (eV) |
| amorphous *a*-Al_84_Fe_16_ | 1138.83 | -1.0 | 1.6 | - | - | - |
| quasicrystals i-Al_62_Cu_25.5_Fe_12.5_ | 1047 | -0.2 | 1.35 | 1.0 | 0.23 | 0.04 |
| i-AlCuFe (1/1) approximant | 477.25 | -0.2 | 0.4 | 0.1 | 0.23 | 0.025 |

**References**

[S1] J. He, L.-D. Zhao, J.-C. Zheng, J. Doak, H. Wu, H.-Q. Wang, Y. Lee, C. Wolverton, M. G. Kanatzidis, and V. P. Dravid, “Role of Sodium Doping in Lead Chalcogenide Thermoelectrics,” *Journal of the American Chemical Society*, vol. 135, pp. 4624, 2013.

[S2] L. Xu, Y. Zheng, J.-C. Zheng, “Thermoelectric transport properties of PbTe under pressure,” *Physical Review B*, vol. 82, article 195102, 2010.

[S3] L. Xu, H.-Q. Wang, J.-C. Zheng, “Thermoelectric properties of PbTe, SnTe, and GeTe at high pressure: an ab initio study,” *Journal of Electronic Materials*, vol. 40, pp. 641-647, 2011.

[S4] N. F. Hinsche, B. Yu. Yavorsky, I. Mertig, and P. Zahn, “Influence of strain on anisotropic thermoelectric transport in Bi2Te3 and Sb2Te3,” *Physical Review B* vol. 84, article 165214, 2011.

[S5] Z. Huang, T.-Y. Lü, H.-Q. Wang, S.-W. Yang, J.-C. Zheng, “Electronic and thermoelectric properties of the group-III nitrides (BN, AlN and GaN) atomic sheets under biaxial strains,” *Computational Materials Science*, vol. 130, pp. 232-241, 2017.

[S6] C. V. Landauro, H. Solbrig, “Modeling the electronic transport properties of Al-Cu-Fe phases”, *Physica B*, vol. 301, pp. 267-275, 2001.
